# *Mycobacterium caprae* Infection of Red Deer in Western Austria–Optimized Use of Pathology Data to Infer Infection Dynamics

**DOI:** 10.3389/fvets.2018.00350

**Published:** 2019-01-21

**Authors:** Annette Nigsch, Walter Glawischnig, Zoltán Bagó, Norbert Greber

**Affiliations:** ^1^Department of Animal Sciences, Quantitative Veterinary Epidemiology, Wageningen University, Wageningen, Netherlands; ^2^Institute for Veterinary Disease Control, Austrian Agency for Health and Food Safety, Innsbruck and Mödling, Mödling, Austria; ^3^Department for Veterinary Affairs, Office of the State Government of Vorarlberg, Bregenz, Austria

**Keywords:** tuberculosis, *Mycobacterium caprae*, red deer, Austria, lesion score, infection dynamics

## Abstract

Austria is officially bovine tuberculosis (TB) free, but during the last decade the west of the country experienced sporadic TB cases in cattle. Free-ranging red deer are known to be the maintenance host of *Mycobacterium* (*M*.) *caprae* in certain areas in Austria, where cattle can become infected on alpine pastures shared with deer. The epidemiology of TB in deer in alpine regions is still poorly understood. To inform decisions on efficient interventions against TB in deer, a method is needed to better capture the infection dynamics on population level. A total of 4,521 free-ranging red deer from Austria's most western Federal state Vorarlberg were TB-tested between 2009 and 2018. *M. caprae* was confirmed in samples from 257 animals. Based on descriptions of TB-like lesions, TB positive animals were categorized with a newly developed lesion score called “Patho Score.” Analyses using this Patho Score allowed us to distinguish between endemic, epidemic and sporadic TB situations and revealed different roles of subgroups of infected deer in infection dynamics. Overall, deer in poor condition, deer of older age and stags were the subgroups that were significantly more often TB positive (*p* = 0.02 or smaller for all subgroups). Deer in poor condition (*p* < 0.001) and stags (*p* = 0.04) also showed more often advanced lesions, indicating their role in mycobacterial spread. TB was never detected in fawns, while hinds were the subgroup that showed the fewest advanced lesions. Analysis of outbreaks of TB and lesion development in yearlings provided some evidence for the role of winter feeding as a source for increased infection transmission. Sporadic cases in TB-free areas appear to precede outbreaks in these areas. These currently TB-free areas should receive particular attention in sampling schemes to be able to detect early spreading of the infection. The Patho Score is a quick, easy-to-apply and reproducible tool that provides new insights on the epidemiology of TB in deer at population level and is flexible enough to relate heterogeneous wildlife monitoring data collected following different sampling plans. This lesion score was used for systematic assessment of infection dynamics of mycobacterial infections.

## Introduction

*Mycobacterium caprae* (*M. caprae*) is part of the *Mycobacterium tuberculosis complex* (MTBC) and is the causal agent of tuberculosis (TB) of cattle and free-ranging red deer (*Cervus elaphus elaphus*) in the border area between western Austria and southern Germany (1–4). In this area, red deer have been identified as TB reservoir that spreads the pathogen through direct or indirect contact to cattle (5). Transmission of TB between wildlife and farmed animals can occur in both directions.

In red deer, TB is a subacute to chronic disease that is associated with emaciation at an advanced stage, but usually does not lead to marked clinical signs (6). TB is commonly diagnosed by presence of lesions in lymph nodes or organs (7). Tonsils are understood to be the main port of entry (8, 9). The medial retropharyngeal lymph nodes drain the tonsils, which is probably the way these lymph nodes become infected (10–12). Accordingly, medial retropharyngeal lymph nodes are often targeted in early detection and monitoring programs (13, 14). As disease progresses within the host, mediastinal and tracheobronchial lymph nodes, lungs, as well as mesenteric lymph nodes can become affected (15). Deer can also show lesions on pleura, in organs within the abdominal cavity, testicles and udder including their regional or subcutaneous lymph nodes (16).

Lesions indicative for TB in red deer range from pinhead-sized to more than 10 cm (in diameter) large granulomas or abscesses. Lesions develop progressively during the subsequent stages of disease and increase in size and number over time. Thin-walled connective tissue capsules containing creamy yellowish-white pus are typical for advanced stages (2, 15–17). These thin-walled abscesses lead in severe cases of generalized TB to high excretion of mycobacteria and thus an increased infectivity of affected animals (6, 18). TB in red deer was reported to be associated with up to 25% of infected animals without macroscopically visible lesions (2, 12). Nugent (19) identified an area in which even 23 (68%) out of 34 culture positive deer had no visible lesions. There are indications that deer that do not die within a year or two of becoming infected can survive for many years (19). Although the detailed pathogenesis of TB in red deer is not fully understood, there is increasing evidence in literature that species-specific stressors, behavioral and environmental factors as well as genetic factors influence susceptibility to mycobacteria (20, 21).

To better understand the development of slowly progressing diseases such as TB on population level, knowledge of the underlying infection dynamics is decisive: when and where did whom spread infection to whom? Especially in the case of wildlife, it is important to exploit all available information to create a valid overall picture and to be able to better target control measures. Another relevant question is the role of subgroups of animals within the deer population for the maintenance and the spread of TB.

This work aims to characterize dynamics of TB transmission within the red deer population to provide evidence for optimized monitoring and control of TB in alpine areas. We also will be investigating whether qualitative and quantitative criteria of TB-like lesions are a suitable indicator to show and measure infection dynamics of TB in deer. On the basis of readily available data, the impact of population structure, time and space will be investigated retrospectively:

- Population structure: do subgroups of animals within the deer population play different roles for the maintenance and the spread of TB?
- Time: did the infection dynamics of TB in red deer in Vorarlberg change between 2009 and 2018?
- Space: are different patterns of infection dynamics observable in the TB zones?

## History of *Mycobacterium caprae* in Red Deer in Vorarlberg, Austria

Austria is recognized as an officially bovine TB-free (OTF) country since 1999. Anecdotal observations suggest that TB was present in deer in the most western Austrian state of Vorarlberg prior to 1999: animals with spherical abscesses of the mesenteric lymph nodes were seen which were later referred to as “ball deer”. But these cases have never been investigated with laboratory diagnostics. The first confirmed TB case in deer in Vorarlberg was recorded in 2006.

**Figure 1:**
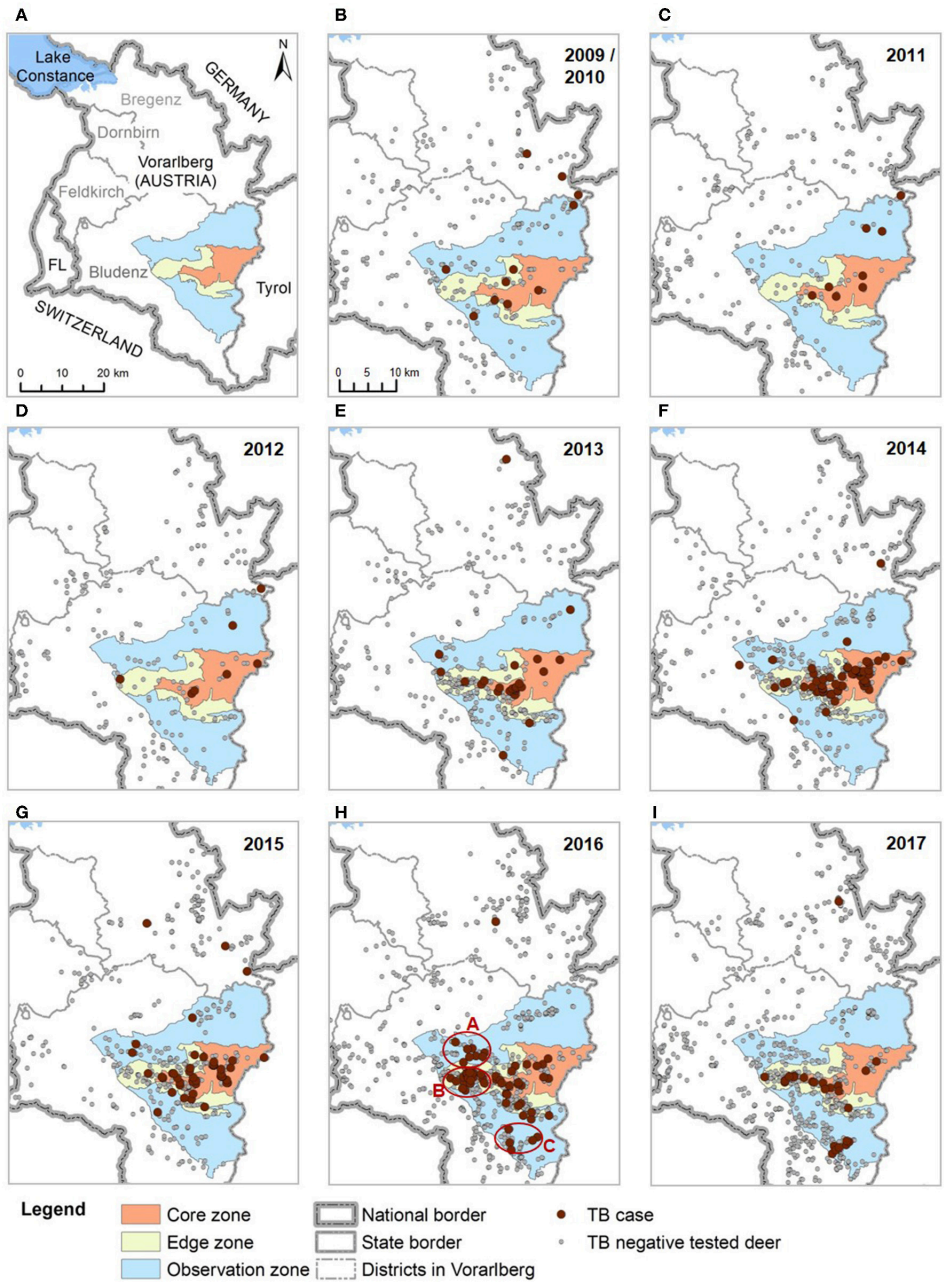
Overview map **(A)** and series of detailed maps of kill locations of TB positive and TB negative deer **(B–I)**, 2009–2017. The core, edge and observation zones form together the TB control area. White areas with kill locations indicate the area outside the TB control area. Cases of the years 2009 and 2010 are shown together in one **(B)**. In 2016 **(H)**, three spots (A–C) with outbreak-like TB are marked in red. FL, Principality of Liechtenstein.

In 2008, TB cases in cattle were reported from the neighboring Austrian state Tyrol, which were linked to infected deer. As a consequence, the first systematic deer monitoring was started in Vorarlberg in 2009 with the aim to assess the risk of TB infection spread to its own cattle population. In the first year of this deer monitoring, *M. caprae* was detected in seven out of a total of 71 examined deer. Since then, TB in deer has been under constant observation. The TB cases are concentrated at a hotspot in two valleys (Klostertal and Montafon north of the river Ill, marked in red as “core area” in Figure 1). About 25–30 km north of this hotspot, TB is detected sporadically in deer in the border area with Tyrol and Germany.

In the alpine areas of Vorarlberg, agriculture mainly consists of small cattle farms with 5–20 animals in extensive farming. A special management practice is the annual transhumance of cattle on alpine pastures above 1,600 m for up to 100 days during summer. During summer, deer also prefer sub-alpine and alpine areas at altitudes up to 2,500 m, where cooler temperatures predominate, and nutrient-rich forage is available. In certain areas, this traditional grazing leads to intensified contacts between deer and cattle. In 2010 TB was also confirmed in cattle in Vorarlberg. In consequence, control measures targeting deer were started in 2011 with intensive hunting under adapted conditions, i.e. the statuary close season (no hunting allowed) was shortened and limits on culling of antlerless animals were abolished in defined areas. Control measures were continuously extended and intensified in parallel to the developments of TB in deer and cattle in order to meet the required increasing total kill numbers in accordance with the official hunting plan.

In deer, TB prevalence seemed to have reached its plateau in 2013 (22). In the TB zone with the highest prevalence (“core zone”), 16 (25%) out of 62 deer samples examined were TB positive in 2013. In the cattle population, TB reached its peak in the winter of 2015/2016 with the detection of *M. caprae* in 30 animals from 13 herds of a total of 9,005 tested cattle from 728 herds (23). In the remaining years between 2013 and 2017, TB was annually confirmed in 4–8 animals from 2 to 7 farms in Vorarlberg (23). The current TB situation does not risk Austria's OTF status yet. The OTF status of a country is based on bovine animals, and a country recognized as OTF will keep this status even though wildlife in the country may be affected, as long as legal conditions are satisfied (Directive 64/432/EEC). However, TB cases in deer require extensive monitoring activities in the cattle population within known deer TB areas. In addition to negative effects on agriculture, hunting and the risk to human health due to this zoonotic agent, annual TB cases led to very high medial and political interest. This interest resulted in part in external pressure for those involved in the control program and reduced their willingness to cooperate in TB control.

### Monitoring Tuberculosis in Cattle and Wildlife

All cattle with potential contact to TB-infected deer are annually examined with the comparative intradermal tuberculin skin test between late November and February, and all animals with non-negative skin tests are culled according to legal requirements. Contact animals are traced, and cattle herds are culled if testing indicates a within-herd prevalence of >40%. In addition, all cattle are inspected for TB at the abattoir as part of the national routine TB surveillance. The combination of these measures aims to reduce the risk of undetected TB cases due to imperfect test sensitivity and to ensure that cattle are TB negative in spring before the start of the grazing period. New TB cases diagnosed in cattle in the subsequent testing period led to the conclusion that the main direction of infection is deer-to-cattle, partly followed by spread from cattle-to-cattle within the infected herd. Spoligotyping (24) and mycobacterial interspersed repetitive unit-variable number of tandem repeat typing (25, 26) confirmed for Vorarlberg that all *M. caprae*-positive deer and cattle tested shared the same genotype “Lechtal” (27).

In addition to cattle and red deer, other wildlife species (badgers, foxes, chamois, roe deer) were tested, albeit not systematically. TB could only be detected in a roebuck in 2017, which was hunted in the known TB area (28). Wild boars are rare in Vorarlberg and have not been sampled to date. Based on current evidence, there is no indication of any significant role of other wildlife species in maintenance of TB infection in wildlife populations or for infection transmission to cattle.

### Red Deer Management in Vorarlberg

Deer hunting is organized by hunting grounds. Deer hunt is seasonal, with a smaller peak in kills in spring and the main kill season in fall. The fall season accounts for two-thirds of the annual hunting bag. In the winter months between end of December to end of March hunting is generally suspended, with the exception of killing of sick or injured deer. An estimated one third of the deer population is hunter harvested each year, with higher percentages in the TB areas due to control measures in place.

A significant cause for establishment and persistence of TB in deer in Vorarlberg is seen in the marked increase in deer densities in certain regions (29). In the 1970s, a change in deer management practices led to a large increase in deer populations far beyond the natural capacity of deer habitats (30). In parallel, extensive developments in land use, such as growth of settlement areas in alpine valleys, the expansion of infrastructure and increase in tourism have reduced habitat of deer. This meant that deer were forced, against their traditions, to spend the winter at higher altitudes. In order to compensate for limited availability of feed and as strategic intervention to protect avalanche protection forests, winter feeding is nowadays carried out during 140 and 200 days a year (31). Winter feeding not only reduces mortality among weakened animals but will also generate artificially high deer densities around feeding sites. Close contact between animals of different age groups supports direct and indirect transmission of TB (32, 33). Winter feeding of deer is still allowed in Vorarlberg. Since 2017, however, there have been restrictions on choice of feed and more elaborate rules for cleaning and disinfecting feeding sites in spring. Additionally, feeding sites are fenced off with cattle-proof fences during the grazing period (34).

Validated information on deer densities over large-scale administrative areas does not exist for Vorarlberg. However, it is known that densities vary largely across alpine regions with considerable seasonal differences: the highest concentration of deer will be recorded around winter feeding sites on harsh winter days with thick snow cover, with focal concentrations of five up to 300 animals on a small number of hectares. In mild winters, groups of deer at feeding sites will be smaller due to availability of natural feed. In summer, deer are distributed over wider areas and groups of deer grazing together are often small (±10 animals) and will rarely reach group sizes of up to 70–100 animals. Radio telemetry studies showed that the summer habitat of deer in alpine areas can be 1.5–4.5 times larger in size compared to the winter habitat (35).

### International Aspects of Infection in Wildlife

Sporadic TB cases in deer in the north of Vorarlberg form a shared deer TB area with Tyrol and Germany (5). In addition, neighboring Switzerland and the Principality of Liechtenstein are at risk of introduction of TB by animal trade and cross-border migration of deer (see Figure 1A, for an overview map). Radio telemetry studies showed that some deer cross the border after the snowmelt, spend the summer in a neighboring country and return to their winter habitat in their “home” country in autumn. Through these migratory individuals the deer populations of Vorarlberg, Switzerland and Liechtenstein are in seasonal contact (35). Deer are monitored for TB both in deer TB areas of Austria and Germany, as well as in TB-free border areas of Switzerland and Liechtenstein. Efforts are made by the four countries to increase comparability of their currently not yet harmonized monitoring programs in order to obtain a transnational overview of the TB situation in deer, as well as to develop a common control strategy (36).

## Materials and Methods

### Study Population and Deer Monitoring

The study population consisted of all free-ranging red deer examined from February 2009 to March 2018 in the deer monitoring in Vorarlberg. The total of 4,521 sampled animals of all age groups were hunter harvested (99.6%) or found dead (0.4%). According to the deer population structure and in line with requirements of deer monitoring, younger and female deer were examined more frequently, with 1,297 (48.2%) deer ≤ 2 years and 2,170 (55.5%) females. A total of 172 (4.0%) animals were in poor condition.

Deer monitoring is carried out in all parts of Vorarlberg with deer habitats and distinguishes four zones corresponding to TB prevalence: the area with highest prevalence is the 103 km^2^ large “core zone”, surrounded by the “edge zone” (77 km^2^) and the “observation zone” (346 km^2^). Core, edge and observation zones form together the 526 km^2^ large TB control area (46.95° N to 47.25° in latitude, and from 09.80° to 10.22° W in longitude) in the district of Bludenz. The fourth zone are the remaining deer habitats in Vorarlberg outside the designated TB control area (1,591 km^2^) where TB has so far been detected only sporadically in deer, mainly in the north in the district of Bregenz (Figure 1). The boundaries of the zones are largely formed by mountain chains and rivers which allow restricted deer movements between zones. Deer abundance is similar in all four zones, with a variation of areas with high and low deer numbers within every zone (37).

Within the 9-year monitoring period, the size of the TB control area and sample size per zone, split by sex and age group, were regularly adjusted depending on case distributions in previous years and published in the annual official deer monitoring program plan (34). Annual sample sizes ranged between 71 and 940 sampled deer. In the hunting season April 2017 to March 2018 all hunter harvested deer except fawns were sampled in core and edge zones (*n* = 211) in accordance with this plan. In the observation zone at least 25% of the hunting bag had to be examined (*n* = 215). Additionally, all deer found dead and sick deer from the whole TB control area had to be investigated. The area outside the TB control area accounted for 401 samples or 20% of the annual hunting bag of deer ≥1 year. The sampled deer do not represent a single random sample.

### Sampling and Diagnostic Methods

Trained hunters checked the deer at the kill location for external abnormalities. Subsequently, thoracic and abdominal cavities of animals were opened, and internal organs examined visually, and partly palpated. If no tissue abnormalities were observed, the standard sampling consisted of lung with its tributary lymph nodes (tracheobronchal and mediastinal lymph nodes) and larynx with medial retropharyngeal lymph nodes (“head and thorax” samples). As the entire hunting bag was sampled in core and edge zones, requirements for sample materials were relaxed for antlerless deer: the tissues to be sampled could be reduced to the head with medial retropharyngeal lymph nodes (“head-only” samples).

From deer with visible tissue abnormalities, the carcass including all internal organs had to be presented for examination to an official veterinarian. Deer found dead and deer in poor condition were as a rule sampled by veterinarians. In addition to standard sample materials, all parts of the carcass with gross lesions were required to be submitted to the Institute for Veterinary Disease Control, Austrian Agency for Health and Food Safety (AGES), Innsbruck. The reality of the given field conditions is that the sampling process and sampled tissues were quite heterogeneous.

Submitted sample material was pathomorphologically examined and all gross lesions were recorded. Lymph nodes with no visible lesions were dissected into 2–4 mm thick slices to detect even small granulomas. Tissue samples with lesions were cultured for 12 weeks at 37°C and MTBC species differentiation was performed by PCR. The analytical protocol to confirm infection with *M. caprae* was described by Fink et al. (5) in detail.

### Development of the Patho Score

To allow spatial-temporal analysis and comparison of the pathomorphological lesion descriptions in free text, a lesion score (“Patho Score”) was developed (Table 1). Based on this score, lesions can be subdivided into six categories (score 0–5) depending on their size, number and distribution in the body. The higher the score, the more advanced stage of TB is observed, with score 0 for non-visible lesions. Each examined animal receives a score for the whole package of submitted sample materials. If the sample material is incomplete, Patho Score tends to underestimate disease progress. The interpretation of the score is based on the hypothesis that TB lesions develop progressively and can be grouped and ordered according to their developmental stage.

**Table 1:** Patho Score for the categorization of TB-like lesions in deer.

**Score**	**Lesion**
0	Non-visible lesion
1	Singular or multiple lesions with < 5 mm in Retro 1^a^
2	Singular or multiple lesions with 5–10 mm in Retro 1^a^
3	Singular or multiple lesions with >10 mm in Retro 1^a^
4	Lymph nodes at multiple body sites affected and/or an organ is affected^b^
5	Overall picture: severe progressed TB/generalization^c^

a *Retro 1, Medial retropharyngeal lymph node with the more advanced lesion. Score 1–3 is based on Retro 1. If both medial retropharyngeal lymph nodes are missing in the sample material, the score for the score levels 1–3 is alternatively based on the lymph node with the most advanced lesion in the submitted sample material*.

b *Retro 1 has score level 3 (>10 mm) and additionally, at least one other lymph node is affected (e.g., Retro 2 with the less advanced lesion, tracheobronchial, mediastinal or mesenteric lymph nodes). By definition, samples with affected organ tissue (lung, pleura, liver, udder, etc.) are categorized at least with score 4, even if the sample material does not contain affected lymph nodes. Reason: According to Cornet's law of localization, the regional lymph node is always affected if the organ is affected (except in chronic organ tuberculosis). Samples consisting only of the head can reach a maximum score of 4*.

c *Example: “Ball deer” with spherical abscessed of the mesenteric lymph nodes, lymphadenitis, lung TB, chronic organ tuberculosis (various organs), severely abnormal lymph nodes*.

The criteria for the Patho Score were: a valid, simple and comprehensible measurement tool with good discrimination, that is able to take into account heterogeneity of sample material and can be applied retrospectively to historical samples. The development of the score was based on a so-called localization principle:

- In deer monitoring, medial retropharyngeal lymph nodes are the only tissues that must be present in all samples, i.e., both in head-only samples (from antlerless deer hunted in core and edge zone) and also in standard head and thorax samples.
- Three score levels (1–3) are based solely on a medial retropharyngeal lymph node (“Retro 1”). The two higher levels (4–5) are based on the overall picture gained from the examination of the entire sample material.
- The size of the lesion has more influence on the level of the score than the number of lesions.

Development of the Patho Score was carried out in several rounds with evaluations by two raters: the pathologist, who had made the pathomorphological assessment of almost all samples, and an epidemiologist. To test the scoring tool, the two raters independently scored 242 TB positive samples based on available historical free text descriptions. The two test results were compared and samples with discrepancies were discussed. After each round, the definitions of the Patho Score were specified with the aim of obtaining the highest possible inter-rater agreement. With the final version of definitions, agreement was reached in 248 out of 257 samples (observed proportionate agreement of 96.5%). Discrepancies occurred with samples of score 4 or score 5, as the definition of score 5 “overall picture of severe progressed TB” is partly subjective. The categorization will thus in a limited number of samples depend on the rating pathologist.

The scoring of the sample takes on average less than 1 min (including documentation). The definitions of the Patho Score are clear and easy to understand. Training of a pathologist who is specifically experienced with TB is therefore considered not necessary.

As addition to the development of the Patho Score, a second pathologist histologically examined a sub-selection of samples in a blinded experiment to verify the character of the macroscopic lesions and to assess feasibility of standardization of scores. This independent evaluation step revealed that confirmation of the pathogen was a prerequisite for inclusion of a sample in the scoring system, since occasionally (especially with mild lesions) other pathogens can cause comparable lesions (e.g., actinomycotic or mycotic granulomas).

TB positive samples that were examined before October 2017 were scored retrospectively. From October 2017 onwards, all fresh samples were scored by the same pathologist on a continuing basis.

### Data Collection and Case Definition

For each sampled animal, data on date of kill event, coordinates and hunting ground of the kill location, age, sex, condition and any further comments by the hunter were recorded in a standardized manner [age groups: males: yearling (1 year), stag III (2–4 years), stag II (5–9 years), stag I (≥10 years), females: yearling (1 year), hind (≥2 years), fawn (from birth till April 1st of following year); condition: good (deer appearing healthy), poor (sick or injured deer with clinical signs)]. Diagnostic results and data on submitted sample materials were recorded by AGES. A central database with all collected data was maintained by the Office of the State Government of Vorarlberg.

Animals were considered a case if *M. caprae* was confirmed by bacterial culture and subsequent species determination. All deer without TB-like lesions or with negative results in bacterial culture were considered negative. In one deer *M. microti* was detected (38), which was classified as (*M. caprae-*) negative in this study. *M. bovis* was never detected.

Inclusion criteria for the analysis were: all deer examined in the deer monitoring with a test result according to the case definition, and presence of a description of the submitted sample material. Excluded were deer that did not meet the minimum requirements for sample material: the sample had to contain at least two of the following lymph nodes or organs: medial retropharyngeal lymph nodes, pulmonary lymph nodes or lung tissue. A total of 4,265 (94.3%) samples met the inclusion criteria (Figure S1). Of these, 334 samples (7.8%) had suspicious lesions. *M. caprae* was confirmed in 257 (6.0%) samples, with 7–72 cases per year. Only positive cases were scored with the Patho Score. Since information on sample material was missing for one case, reported results are based on 256 of the 257 confirmed *M. caprae* cases.

### Data Analysis

The descriptive analysis of the spatial-temporal development of the Patho Score over a period of nine years and the statistical association between animal-specific risk factors for TB status and Patho Score of advanced TB-like lesions were carried out in STATA (39) using Pearson's chi-squared test, Cochran–Mantel–Haenszel test (MH) and Wald test of homogeneity of stratum-specific odds ratio's (OR). The Patho Score was used as an indicator to systematically show and quantify dynamics of infection. For comparisons of mean Patho Score between subgroups, the arithmetic mean of scores was calculated (reported with the 95% confidence interval (CI)). For the adjusted MH test, the Patho Score was reduced to two levels (low scores: 1–3; and high scores 4–5).

For the MH test, the reference categories were fawns (vs. yearlings and adults >2 years for the variable “age”), males (vs. females for the variable “sex”), good condition (vs. poor condition for the variable “condition”), head-only samples (vs. additional sample tissues for the variable “sample tissue” type) and zone outside the TB control area (vs. observation zone, edge zone and core zone for the variable “zone”). For the binary variable sample tissue “head and thorax,” “head, thorax and abdomen,” “thorax-only,” and “other” samples were subsumed under samples with “additional tissue” (Table 2). For the variable zone, the score test for trend of odds was applied; the reported OR estimate is an approximation to the OR for a one unit increase in the level of zone).

**Table 2:** Body sites examined and location of TB-like lesions.

**Body sites examined**	**Lesion location**						**Total (%)**	
	**Retro**^**a**^ **(%)**		**Thorax (%)**		**Other tissue (%)**			
Head-only	115	(98.3)	–	–	4	(3.4)	117	(45.7)
Head and thorax	96	(93.2)	24	(23.3)	1	(1.0)	103	(40.2)
Head, thorax and abdomen	11	(57.9)	11	(57.9)	12	(63.2)	19	(7.4)
Thorax-only	–	–	6	(100)	–	–	6	(2.3)
Other	5	(45.5)	4	(36.4)	3	(27.3)	12^b^	(4.3)
Total	227	(88.7)	45	(17.6)	20	(7.8)	256	(100)

*Row percentages of lesion locations may exceed 100% due to lesions at multiple locations in the sample material of an animal. The last column presents column percentages*.

a *Medial retropharyngeal lymph nodes (present in 248 cases, affected in 227 cases)*.

b *Only affected sample material was described, but overall information on submitted tissues was missing*.

**Figure 2:**
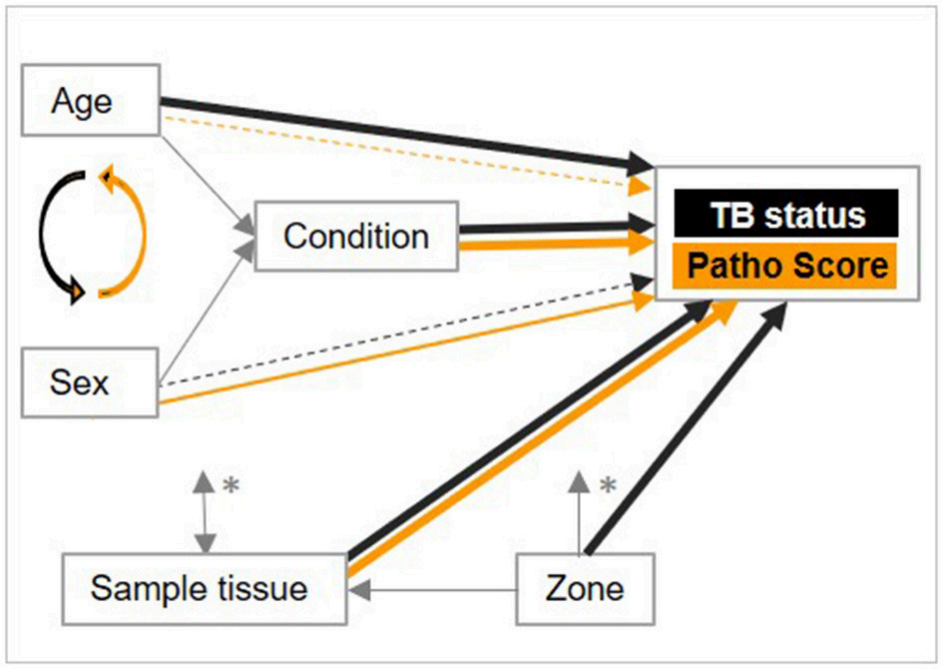
Causal diagram of links between five explanatory variables with the outcomes “TB status” and “Patho Score.” Black arrows: links with TB status. Orange arrows: links with Patho Score. For black and orange arrows, thicker arrows indicate stronger evidence for an association with the outcome. Dotted arrows indicate that only one level of the variable appears to be associated with the outcome. Curved arrows indicate interaction between variables. Gray arrows link explanatory variables with each other without any assumptions regarding strength of evidence of an association. ^*^Sample tissue type, age, sex, and condition influence each other in both directions. Zone influences age, sex and condition.

A causal diagram was used to conceptualize links between the three animal-specific *in vivo* recordable variables age, sex, condition, and the two further explanatory variables sample tissue and zone with the outcomes “TB status” and “Patho Score” and for bias assessment (Figure 2). Condition was identified as an intermediate variable on the causal path between both age and sex with TB status and with Patho Score. Age and sex were thus not adjusted for condition to avoid overadjustment. Zone influences age, sex, condition and sample tissue type through zone-specific differences in the sampling within the deer monitoring. The number of sampled tissues is influenced by age, sex and condition according to the deer monitoring program plan, but also influences the chance that an individual of a certain age, sex, or condition becomes a case, or receives a high Patho Score.

The spatial data visualization and analysis was done in ArcGIS (40). Analyses with annual comparisons are based on the official period of the hunting year (April 1st–March 31st), e.g., 2017 includes all deer tested between April 2017 and March 2018. February and March 2009 were counted to the hunting year 2009.

## Results

### Submitted Sample Material

In a total of 117 (45.7%) out of 256 cases the submitted sample material consisted only of the head or parts of the head including medial retropharyngeal lymph nodes (“head-only,” see Table 2); 60 (51.3%) of these samples were obtained from hinds and 41 (35.0%) from yearlings. The second largest group were 103 (40.2%) samples consisting of head and thoracic organ tissues (“head and thorax”). See Table S1 for detailed numbers of deer tested, split by subgroup, TB status and Patho Score.

In 227 (91.5%) out of the 248 samples containing at least one medial retropharyngeal lymph node, this lymph node was affected. Lung or pulmonary lymph nodes were affected in 45 (32.3%) out of 139 samples containing thoracic organ tissues). Other sample tissues with TB-like lesions (“other tissues”) comprised parts of the intestine with mesenteric lymph nodes, liver with hepatic lymph nodes, diaphragm with pleura and udder tissue including mammary lymph nodes. Since other tissues were to be presented only in case of visible abnormalities, no valid conclusion can be drawn from these data regarding true frequency of lesions in abdominal organs or other body parts, but they give an overview of the range of lesions and organs affected.

### Pathomorphology of Lesions

The pathomorphological abnormalities of TB-like gross lesions corresponded to earlier descriptions on *M. caprae* in red deer in western Austria (4, 5, 16, 41). It could be confirmed that observed lesions predominantly consisted of granulomas and abscesses with creamy pus or caseous cores. With Patho Score 1, lesions were mostly singular, 1–5 mm large granulomas or micro-abscesses in a single lymph node. In 47 (97.9%) of a total of 48 samples with score 1, one or both medial retropharyngeal lymph nodes were affected. Only in one sample, the medial retropharyngeal lymph node itself showed no alterations, but had a 3 mm abscess of creamy-yellowish pus in its immediate vicinity. The exact localization of this lesion could not be identified due to the conduct of the sampling.

Lesions with score 2 were characterized by singular or multiple abscesses with 5–10 mm of diameter, with creamy, purulent-watery or caseous contents, some of which were encapsulated. Score 3 lesions were grossly similar to lesions described for score 2, but with coalescing abscesses that formed singular abscesses with diameters of up to 80 mm. Lesions with score 4 showed a more differentiated picture: in addition to the increasing sizes of typical abscesses in the lymph nodes, numerous miliary granulomas were observed in lymph nodes or the lungs. Lymph nodes were in some cases very small and of firm consistency. Lesions with score 5 corresponded to generalized TB with advanced lesions in multiple lymph nodes and organs with abscesses up to 200 mm in diameter (Figure S2).

Cases with head-only samples received a mean score of 2.4 ± 0.2, and cases with additional tissues had a mean score of 3.2 ± 0.2. Within the group of cases with additional tissues mean scores did not differ significantly after adjustment for condition: for animals in poor condition all carcass parts with gross lesions had to be submitted, leading to more sampled tissues with higher numbers of gross lesions. However, among 19 cases with the maximum range of sampled sites (head, thorax and abdomen, Table 2), only one case would have received a lower Patho Score if only the standard sample (head and thorax) would have been presented for pathological examination. This was the only case with TB lesions in the mesenteric lymph nodes but without gross lesions in the medial retropharyngeal lymph nodes or thoracic tissues.

### Risk Groups for TB Positivity

**Figure 3:**
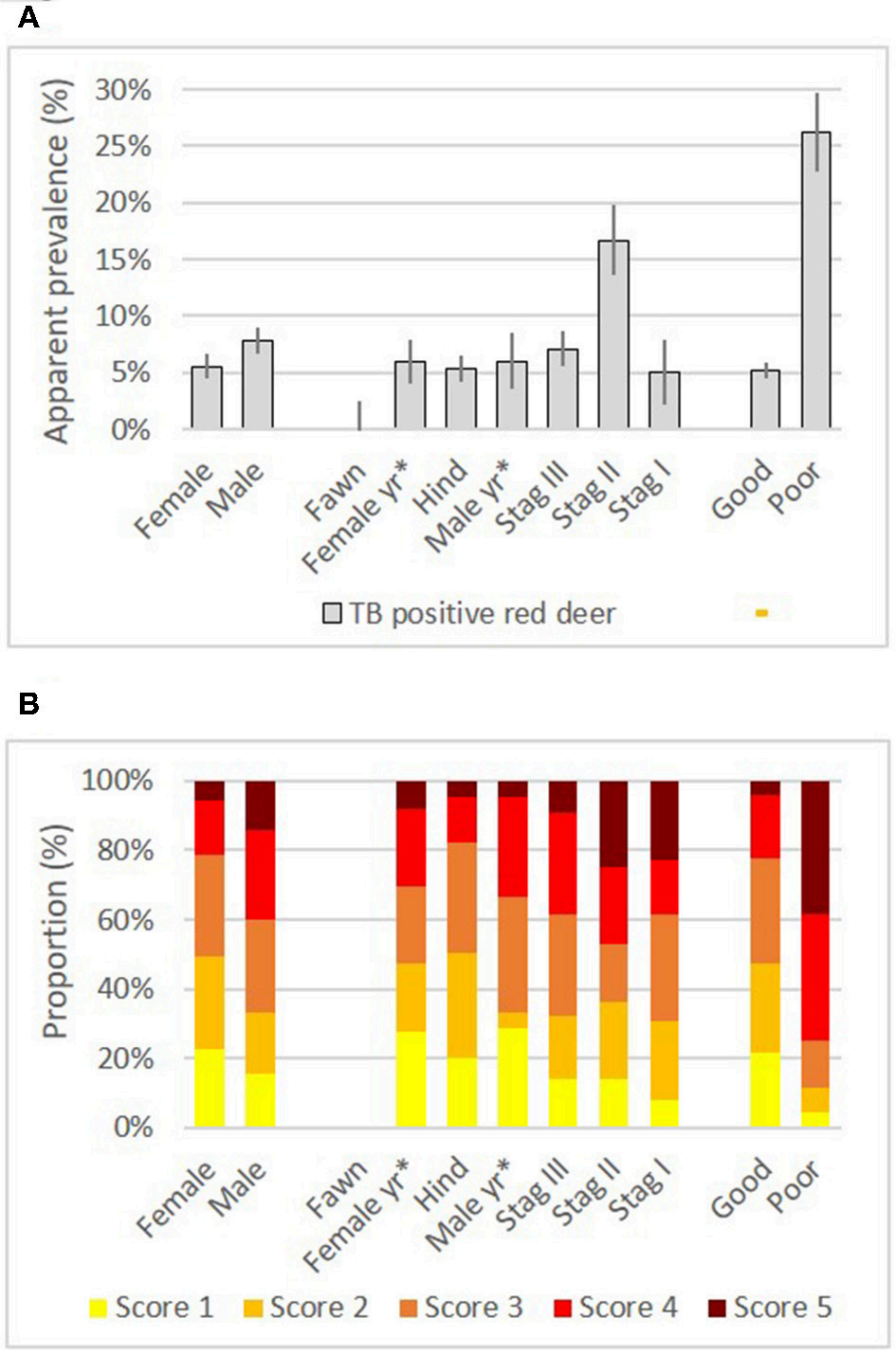
Apparent prevalence with 95% confidence intervals **(A)** and distribution of Patho Scores **(B)**, by sex, sex & age group and condition of TB positive deer (*n* = 256). For better comparability of groups, the Patho Score was normalized in **(B)**. Example how to read the panels: 5.6% of all females were tested TB positive, and of these, 22.3% were categorized with score 1, etc. No sex was recorded for fawns. ^*^yr, yearling, hind: ≥2 years, stag III: 2–4 years, stag II: 5–9 years, stag I: ≥10 years.

Figure 3A shows the (crude) apparent prevalences for deer subgroups split by sex, age and condition. Table 3 list detailed statistical output for this chapter. In the crude analysis the MH chi-squared test showed very strong evidence (*p* < 0.001) for associations between TB status and the explanatory variables sex, age, condition, zone and sample tissue type. In pairwise adjustments against each other, the MH analysis confirmed the strength of association between condition, zone and the sample material and TB status: deer in poor condition had 6.5 times the odds of having TB. For zone, the score test for trend showed an OR of 2.6 for a one unit increase in zone, with the area outside the TB control area as baseline. The crude OR of 0.6 for sample tissue type was confounded by the differing sampling method in the TB zones. In the low prevalence zone outside the TB control area, only 5.1% of submissions were head-only samples. In the observation, edge and core zones, the percentages of head-only samples were 39.0, 66.1, and 62.7%, respectively. After controlling for zone, deer with additional submitted sample tissues had 1.8 times the odds of TB positivity compared to deer with head-only samples.

**Table 3:** Models selected to explain the association between TB status and age, sex, condition of deer, number of submitted sample tissues and TB zone of kill location.

**Explanatory variable for TB status**	**Adjusted for**	***n* (cases)**	**chi^**2**^**	***p*, MH**	**OR**	**95% CI**	***p*, Wald test**
Age	Crude MH^a^	4,262^b^ (257)	18.50	< 0.001	1.55	1.27–1.89	
	Zone, sample tissue	3,982^b^ (257)	32.87	< 0.001	1.90	1.53–2.39	0.94
Age (males only), in area^c^	Sample tissue	1007^d^ (125)	6.63	0.01	2.00	1.17–3.45	0.17
Age (females only), in area^c^	Sample tissue	1,296^d^ (118)	3.94	0.05	1.52	1.00–2.30	0.78
Sex	Crude MH^a^	3,912 (257)	7.83	0.005	1.43	1.11–1.85	
Sex, outside area^c^	Age	1,611 (14)	5.97	0.01	4.54	1.19–17.16	0.10
Sex (yearlings only), in area^c^	Sample tissue	746 (56)	0.02	0.88	1.04	0.59–1.85	0.94
Sex (adults only), in area^c^	Sample tissue	1,446 (187)	5.05	0.02	1.53	1.05–2.20	0.02^e^
	Head-only	667 (75)	0.27	0.61	0.85	0.47–1.55	Stratum 1^e^
	Additional tissue	779 (112)	10.04	0.002	2.16	1.33–3.52	Stratum 2^e^
	Sample tissue, condition	1,446 (187)	3.14	0.08	1.41	0.96–2.05	0.12
Condition	Crude MH^a^	4,264 (257)	128.27	< 0.001	6.48	4.47–9.41	
	Zone, sample tissue	3,983 (257)	100.46	< 0.001	6.62	4.32–10.15	0.12
Sample tissue	Crude MH^a^	3,983 (257)	14.76	< 0.001	0.56	0.47–0.79	
	Zone	3,983 (257)	16.84	< 0.001	1.76	1.36–2.41	0.31
Zone	Crude MH^a^	4,265 (257)	289.42	< 0.001	2.64	2.36–2.95	

*For each explanatory variable, the table presents the variables adjusted for, number of independent samples (and cases thereof), (pooled) chi-squared statistic, p-value and estimate of the odds ratio of the Cochran-Mantel-Haenszel (MH) test with 95% confidence interval, and p-value of the Wald test for homogeneity of the odds ratios of the stratified analysis. All tests have one degree of freedom*.

a *Crude MH: MH analysis without adjusting for other variables*.

b *Age in three categories: fawns (reference)—yearlings—adults ≥2 years*.

c *Area: TB control area, consisting of core, edge and observation zones*.

d *Age in two categories: yearlings (reference)—adults ≥2 years, as sex was not recorded for fawns*.

e *Stratum-specific odds ratios need to be reported*.

The Wald test and the comparison with MH adjusted OR for this bivariate analysis suggested sample tissue type and zone as potential confounders for the association between sex and age with TB status. These confounders were thus controlled for in the following analyses. Sex proved to be a weak indicator for TB status**:** only for the area outside the TB control area, the adjusted MH estimate showed good evidence (*p* = 0.01) for an association between sex and TB status: males had 4.5 times the odds of being TB positive. In the TB control area, age appeared to modify the effect of sex on TB status (and *vice versa*): there was no difference between male and female yearlings (*p* = 0.88). But for adult deer the analysis showed good evidence for an association (*p* = 0.02) between sex and TB status: stags had 1.5 times the odds of being TB positive compared to hinds. The estimated OR was larger for adults with additional sample tissues (OR = 2.2). This association was to a large extent explained by the fact that stags (especially stags II) were more often sick or injured. Out of the 39 cases in adult deer with poor health, 31 (79.5%) were male. After additional adjustment for the intermediate variable condition, stags had 1.4 times the odds of being TB positive compared to hinds (*p* = 0.08). There was thus only weak statistical support for a controlled direct causal effect of sex *per se* on TB status.

Age *per se* showed to be a good indicator for TB status: After adjusting for zone and sample tissue type, there was even stronger evidence (*p* < 0.001) for an association between age with TB status (adjusted OR = 1.9 vs. crude OR = 1.6). Stratified analysis by sex showed that the odds for TB positivity increased in both sexes with age: stags had 2.0 times the odds of TB positivity compared to male yearlings, and the odds of hinds were 1.5 compared to female yearlings. Out of all subgroups split by sex and age, 5–9 year old stags II showed the highest apparent prevalence of 16.7% (Figure 3A). None of the 351 tested fawns was tested TB positive.

Condition was found *per se* to be the most important *in vivo* recordable indicator for TB status. Emaciated, sick or injured deer had after adjusting for zone and sample material around 6.6 times the odds of being tested TB positive compared to deer appearing healthy (*p* < 0.001).

### Risk Groups for Advanced Lesions

Figure 3B shows the distribution of the Patho Score for deer subgroups split by sex, age and condition. Table 4 list detailed statistical output for this chapter. Comparing the crude means of the Patho Score (with levels 1–5), hinds had the lowest mean score (2.5 ± 0.3), followed by yearlings (females: 2.6 ± 0.4; males 2.8 ± 0.5) and stags (stags III: 3.0 ± 0.3; stags II: 3.2 ± 0.4 and stags I: 3.2 ± 0.7). The crude MH analysis showed very strong evidence (*p* < 0.001) for an association between Patho Score (reduced to two levels high/low) and condition and sample tissue type, and strong evidence (*p* = 0.002) for an association between Patho Score and sex. Deer in poor condition, deer with additional sample tissues and males had 10.5, 3.0, and 2.4 times the odds of having a high Patho Score, respectively. There was no evidence for an association between Patho Score and age or zone in the crude analysis.

**Table 4:** Models selected to explain the association between Patho Score and age, sex, condition of deer, number of submitted sample tissues and TB zone of kill location.

**Explanatory variable for Patho Score**	**Adjusted for**	***n***	**chi^**2**^**	***p*, MH**	**OR**	**95% CI**	***p*, Wald test**
Sex	Crude MH^a^	256	10.14	0.002	2.44	1.38–4.29	
	Sample tissue	256	2.01	0.16	1.55	0.84–2.87	0.17
Sex (yearlings only)	Sample tissue	57	0.05	0.83	0.87	0.25–3.04	0.65
Sex (adults only)	Sample tissue	199	4.25	0.04	2.15	1.02–4.54	0.14
Age^b^	Crude MH^a^	256	0	0.95	0.98	0.52–1.85	
Age^b^ (males only)	Sample tissue	135	0.09	0.77	1.17	0.40–3.40	0.31
Age^b^ (females only)	Sample tissue	121	3.51	0.06	0.40	0.15–1.08	0.62
Condition	Crude MH^a^	256	47.15	< 0.001	10.53	4.55–24.36	
	Sample tissue	256	35.57	< 0.001	9.43	3.92–22.69	0.35
Sample tissue	Crude MH^a^	256	14.84	< 0.001	3.02	1.67–5.46	
Zone	Crude MH^a^	256	1.59	0.21	0.83	0.62–1.11	

*For each explanatory variable, the table presents the variables adjusted for, number of independent samples (and cases thereof), (pooled) chi-squared statistic, p-value and estimate of the odds ratio of the Cochran-Mantel-Haenszel (MH) test with 95% confidence interval, and p-value of the Wald test for homogeneity of the odds ratios of the stratified analysis. All tests have one degree of freedom*.

a *Crude MH: analysis without adjusting for other variables*.

b *Age in two categories: yearlings (reference)–adults ≥2 years, as TB was never detected in fawns*.

Sex appears to be a good indicator for advanced lesions in adult deer: Like with TB status, the Wald test indicated interaction between age and sex in respect to their effect on the Patho Score. Adjusting for sample tissue type showed for yearlings no evidence for an association between sex and Patho Score (*p* = 0.83). For adults however, there was good evidence for an association (*p* = 0.04). Stags had 2.2 times the odds of having advanced lesions compared to hinds.

Age is a weak indicator for score 4–5 lesions: Stratified by sex and adjusted for sample tissue type, there was no statistical support for an association between age and Patho Score with males (*p* = 0.77), although the percentage of advanced lesions increased tendentially with age (except the oldest age group of stags I). Out of all age groups of males, stags II were with 17 (45.9%) of 37 submissions the subgroup with the most lesions with scores 4–5. Females showed an opposing trend: there was some evidence for an association between age and Patho Score (*p* = 0.06). Hinds had 0.4 times the odds, or, in other words, female yearlings had 2.4 times the odds of having a high score compared to hinds. Zone did not confound the association between sex and age with Patho Score; the ORs adjusted for zone did only marginally differ from the ORs adjusted only for sample tissue type (results not shown).

Condition was again found to be the most important indicator for advanced lesions with scores 4–5. Independent from the levels of age, sex, tissue material or zone, deer in poor condition had 9–10.5 times the odds of showing advanced stages of TB (result shown in Table 4 are limited to crude analysis and adjustment for sample tissue type). Clinical signs or other abnormalities were recorded for 45 (26.2%) cases; of these 33 (73.3%) received score 4 or 5 (Figure 3B). Emaciation was with 12 records the most frequent leading symptom. For another nine deer leg injuries or other injuries were reported. For most cases only non-specific records on the clinical signs were available (“sick,” “abnormal behavior”). In general, stags II contributed most to the subgroup of deer in poor condition (19 (42.2%) of 45 cases).

### Infection Dynamics in the Years 2009–2017

**Figure 4:**
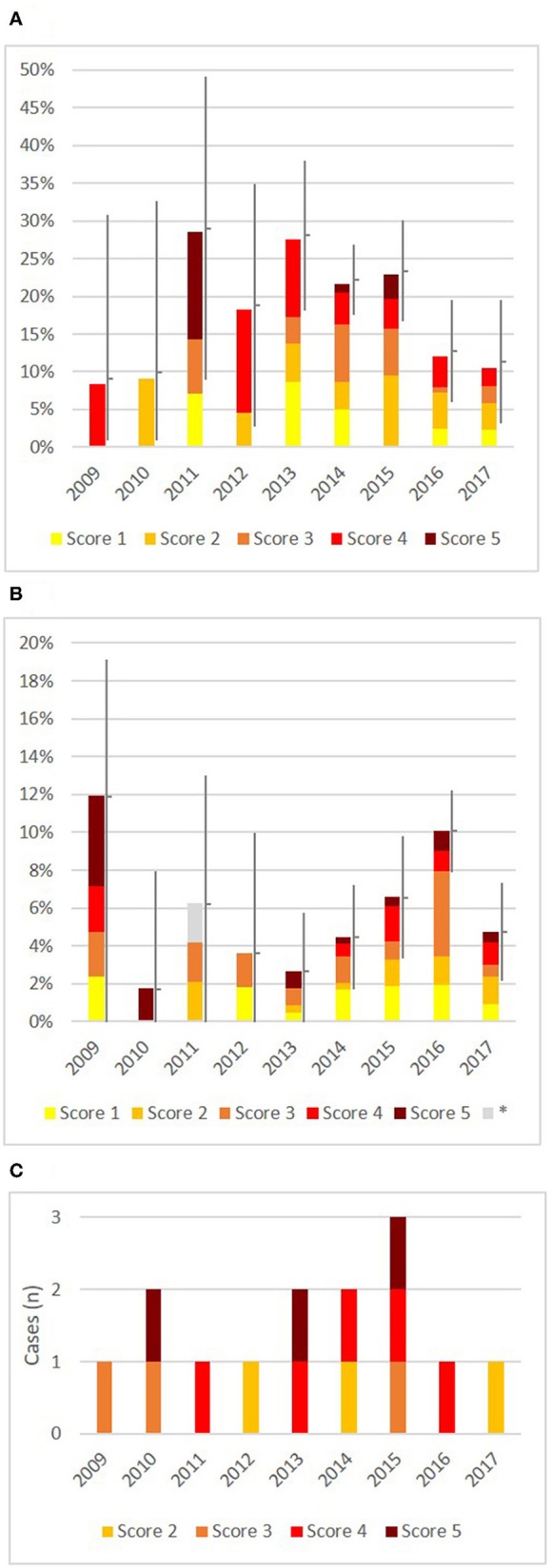
Development of the Patho Score between 2009 and 2017, stratified by TB zones. **(A)** Core zone with in total 136 TB positive deer out of 719 tested deer. **(B)** Edge zone and observation zone (joined) with in total 106 TB positive deer out of 1,733 tested deer. ^*^Data on submitted material was missing for one case marked in grey, therefore no score was assigned. **(C)** Area outside the designated TB control area with in total 14 TB positive deer out of 1,815 tested deer. Colors of cases denote the Patho Score. In **(A,B)**, the bars mark the apparent prevalence with 95% confidence intervals; in **(C)** the number of sporadic cases is shown. Up to 2012, the confidence intervals are large due to lower sample size.

Geographically, the distribution of TB cases in the core, edge and observation zones remained relatively constant between 2009 and 2012 (Figures 1B–D). From 2013 onwards, a redistribution of cases took place: while apparent prevalence decreased in the core zone since 2013, it increased in edge and observation zones in 2013–2016. In the core zone, apparent prevalence was significantly lower (*p* < 0.03) in 2016 and 2017 with 12.0 (±6.8%) and 10.6% (±8.3%) respectively, compared to 2013–2015 with 21.6–27.6% (±4.7–10.0%) (Figure 4A). In the neighboring edge and observation zone apparent prevalence was with 10.1% (±2.2%) in 2016 also significantly higher (*p* ≤ 0.002) than in 2013 (2.7% ± 2.7%), in 2014 (6.6% ± 3.1%), and also in 2017 (4.8% ± 2.5%). This development was comparable in edge and observation zones and therefore both zones are presented in a joint graph in Figure 4B. TB has noticeably spread since 2013, especially in the west and the south of edge and observation zones (Figures 1E–H). See Table S2 for detailed statistics related to apparent prevalences.

Three different patterns of disease occurrence could be identified: endemic disease, epidemics and sporadic cases. These three patterns will be described in more detail.

#### Endemic Disease

Analyses of the Patho Score showed that all stages of TB occurred together in the core zone. This corresponds to the typical picture of an endemic disease occurrence without much tendency of a change. From 2013 onwards, proportions of all score levels decreased at a fairly similar scale along with a decreasing apparent prevalence (Figure 4A) (no *p*-value reported due to several subgroups with zero individuals). There was still evidence for an active infection cycle in 2016 and 2017, which is revealed by 2% of tested deer with score 1, including yearlings. However, deer with score 5 were missing in sample materials in these last two years with lower apparent prevalences.

#### Epidemic in a Newly Infected Area

The increase of prevalence was no zone-wide evenly distributed phenomenon but was attributable to three newly infected spots in the edge and observation zones that were confirmed in 2016 (Figure 1H shows spots A–C). The 2016 hunting year had both the highest apparent prevalence (10.1% ± 2.2%), and also the highest proportion of higher scores (7% of deer with scores 3–5) recorded so far in these two zones.

**Figure 5:**
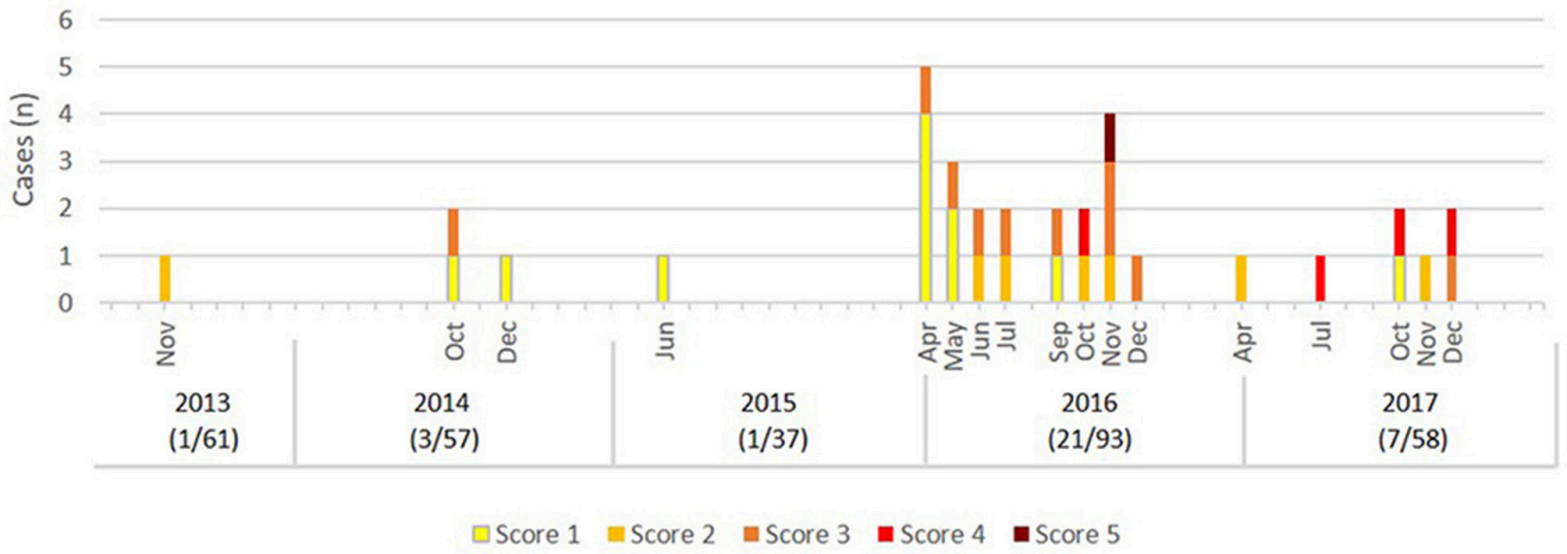
Epidemic curve of TB cases in deer by month of detection for the years 2013–2017, spot B. Colors of cases denote the Patho Score. In Brackets: (number of cases/number of deer tested per year).

Spot B will be described as example for epidemic TB in more detail: This spot was a 23 km^2^ large hunting ground located west of the core zone in the edge zone. In 2013, a first case of TB was detected in a female with score 2 (Figure 5). In 2014, three animals were positive (all three were females with scores 1 or 3), followed by a case of a yearling with score 1 in 2015. In 2016, five cases were shot right at the beginning of the hunting season in April. This unexpected finding led to an intensified hunting and sampling of deer in this area and resulted in a total of 21 cases out of 93 tested deer. The infection could first be detected in yearlings and females in spring and summer. Only from October 2016 onwards scores 4 and 5 were found (in stags). The first deer with clinical signs was a stage II with score 5 in November.

In two outbreak-like spots in the north-western (spot A) and southern observation zone (spot C), TB was confirmed in 2016 with seven and five cases respectively (Figure 1H). In both spots the first cases were also detected in March and April. In spot A, the first case was a stag III with score 1, followed by cases with scores 2 and 3 and 11 month later one case with score 5 (no cases detected in 2017). In spot C, the first case was a female yearling with score 5, followed by cases with scores 1 and 3, and seven more cases in 2017 presenting lesions of all five levels of scores.

#### Sporadic Cases Outside the Designated TB Control Area

**Figure 6:**
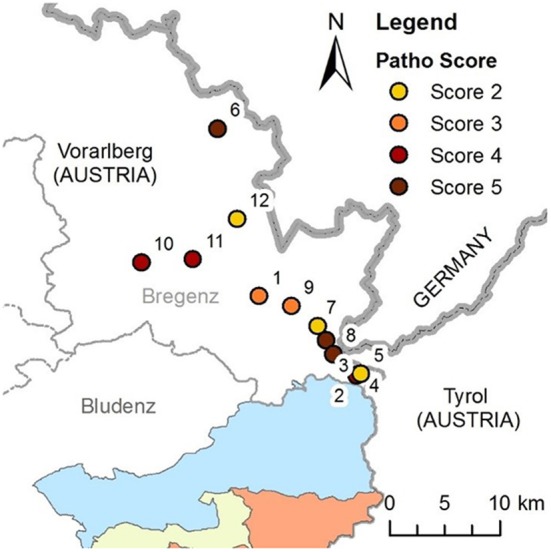
Map of kill locations of 12 TB cases in deer in the area outside the designated TB control area, district of Bregenz. Zones of the TB control area are marked in blue, yellow and red. Case numbers are sorted by kill date: 1: earliest sample from August 2009, 12: latest sample from September 2017). Colors of cases denote the Patho Score (Table S3 lists the characteristics of cases).

Between February 2009 and March 2018, a total of 14 TB cases were detected outside the designated TB control area, with one to three cases each year (Figure 4C). Twelve of these cases were recorded in the district of Bregenz (Figure 6). Age and sex distribution among these cases showed with nine males and only one yearling a different pattern compared to the TB control area (Table S3). With eight (57.0%) cases with score 4 or 5 lesions, advanced TB stages were frequent, and score 1 lesions were not detected so far. Kill locations were up to 30 km away from each other and in different valleys. On the one hand, cases appeared to be independent in time and space. On the other hand, patterns are visible: all deer were hunted between August and November, and kill locations of eight of the 12 cases lie on an imaginary line (cases numbers 1–5 and 7–9, see Figure 6).

## Discussion

This study attempts to describe infection dynamics of TB in red deer by using patho-scoring as an additional source of information. The study demonstrates that the infection dynamics of TB are associated with individual animal-specific parameters such as sex, age and condition, and environmental characteristics such as vicinity to other TB areas.

### Roles of Subgroups in the Infection Dynamic

Three animal-specific parameters are usually recorded by the hunter before the kill: age, sex, and condition. Older animals and stags were significantly more likely to be TB positive. Stags also had higher Patho Scores than hinds. The subgroups with the lowest apparent prevalence were fawns; and hinds had on average the lowest scores. Effect modification between age and sex on their observed effect on TB status and the developmental stage of lesions could indicate that stags, hinds and yearlings play different roles in the infection dynamics within the infected deer population. These roles for the spread and maintenance of disease of different subgroups should be investigated in more detail to better understand how TB spreads within and between deer populations.

The majority of studies report higher TB prevalence in male deer [reviewed by (42)], including studies from Spain and Michigan (32, 43, 44). In contrast, similar prevalence values were recorded in male and female deer in New Zealand, with a higher prevalence in young males ≤2 years offset by a lower prevalence in older males > 5 years (19). Lugton et al. (12) observed more gross lesions and more cases of advanced TB in male than female red deer, although this difference was not significant. They interpreted this finding not as a higher susceptibility of males to TB *per se*, but attributed this result to the time the stags were shot, which usually fell into the period during which males were in hard antler. During rutting season, stags experience particular stress from aggression and gathering and maintaining a group of hinds, which may influence the development of lesions (12). The results of this study give some support to this conclusion: after adjusting for the (intermediate variable) condition, there was only weak statistical support for a controlled direct causal effect of sex on TB status. Higher prevalences of TB and advanced lesions in stags were largely attributable to higher proportion of sick or injured deer among males compared to hinds.

TB was not detected in fawns in this study. The observation of no or very low numbers of TB positive fawns has been made on several continents (32, 44, 45) and has been interpreted as indication for the limited importance of dams for the infection of their fawns in free-ranging deer. Conversely, for farmed deer, Griffin et al. (46) described an acute outbreak of TB with a prevalence >90% in young fawns accompanied with a prevalence of 60% in breeding hinds. One feasible explanation would be that the TB status of fawns is indeed directly related to the level of exposure from hinds. In Vorarlberg, hinds were one of the subgroups with the lowest prevalence and also had the lowest Patho Scores. The absence of infection in fawns despite 5% of their mothers being infected could also indicate that direct deer-to-deer transmission is very rare, and fawns are only infected in settings with significant indirect environmental transmission. This might not happen in Vorarlberg until winter feeding begins.

Higher figures of infection in older deer are a common finding in TB literature and are attributed i.a. to long exposure time of this long-lived species and chronicity of TB (12, 44, 47). In Vorarlberg, the group of 5–9 year old stags II had both the highest prevalence of TB positivity and advanced lesions. Over 10 year old stags I were three times less likely to be TB positive compared to stags II, and also less often showed generalized TB (Figure 3). The observation that gross lesions are less frequently detected in very old animals was also made by Lugton et al. (12). The authors hypothesized that infected animals may be capable of resolving lesions over time or that susceptible individuals have died, while those remaining have kept the infection under control.

These two different tracks of disease progression could also serve as explanations for the special role of the oldest deer in Vorarlberg: the high numbers of stags II with clinical signs and advanced TB would correspond to the susceptibles that are killed sick or die before they reach the oldest age group. For stags that reach apparently healthy an old age, although they might have been exposed to the pathogen for years, this could indicate some form of natural immunity or clinical latency. An additional explanation could lie in distinct contact patterns. It has been observed that older stags prefer to roam in very small groups of stags or sometimes even become solitary. Such behavior could lead to a limited exposure to the pathogen. To conclude, in case a protective factor could be identified, it would be relevant to investigate whether those “protected” subgroups also show lower infectivity and whether the *in vivo* identification of these animals could be utilized within a selective TB control strategy.

The most important *in vivo* indicator for TB was however the condition. Cases with poor health also had advanced stages of TB (score 4 or the maximum score 5) significantly more often. This relationship was to be expected since the observed clinical signs were likely to be attributable to the disease progress of TB in a considerable number of cases. Research showed that deer with advanced TB can cause massive environmental contamination through the excretion of high numbers of mycobacteria (8, 12, 48). The findings of this study suggest that selective culling that aims at the elimination of potential high shedders should prioritize weak, sick and injured deer, even if no abnormalities indicative for infectious disease are observed. However, to increase the efficiency of a control strategy that includes selective culling, it is advisable to combine findings on risk groups with additional epidemiological information to identify groups of deer with higher exposure or locations with increased risk of environmental contamination, e.g. by targeting groups or areas with earlier detections of deer with scores 4–5.

### Patterns of Disease Occurrence

Three different patterns of TB occurrence could be distinguished and characterized: areas with endemic disease, areas with outbreak-like cases, and areas with sporadic cases. This distinction is relevant to assess the infection dynamics in each area and to better inform the selection of targeted prevention and control measures.

#### Endemic Disease

In the core zone both new infections as well as spreaders with advanced TB were seen, which can lead to further infections. This suggests that an endemic equilibrium has been reached with multiple infection chains occurring in parallel. Surprisingly, only few deer with score 5 were detected in the core zone. In this zone, deer have been intensively hunted for several years as part of TB control and apparent prevalence is declining. The question remains whether absence of cases with score 5 is causally associated with the decline of the apparent prevalence. To exclude biases due to sampling regime or to confirm other potential reasons these developments need to be further monitored.

#### Epidemic Disease

Outbreak areas showed the typical picture expected for a point source in a previously disease-free population: the first detected cases presented predominantly early stages of lesions. Over time, more advanced disease stages and cases of generalized disease (score 5) were seen, which eventually were accompanied by clinical signs (Figure 5). A characteristic of a point source is that all cases are exposed at one point in time or within a limited period of time and location directly or indirectly to the primary case. Whether the pathogen was introduced by a single “super spreader” or by multiple animals serving as co-primary cases cannot be distinguished from the data. Due to the proximity of outbreak areas to endemically infected areas both scenarios are possible.

In all three outbreaks spots first cases were detected in early spring, pointing toward infection spreading during the winter feeding season. In spring, deer tend to browse still close to the location of their feeding site. It would need to be investigated if through early detection of cases in spring deer belonging to the same winter feeding cohort can be identified. Such a classical approach of tracing back could support targeted hunting of deer at higher risk of infection and thereby prevent that TB establishes permanently in a new area. Whether the three outbreak spots already reached a state of endemic infection or whether control measures were successful in limiting further spread will become clearer within the next 1–2 years. Within one year of detection of the first cases both early and advanced stages of lesions have been found in the three outbreak spots. The case of a yearling killed in April with score 5 indicated that TB can quickly progress to stages where infected individuals may cause massive environmental contamination.

#### Sporadic Cases

Sporadic cases should receive particular attention: so far, all outbreaks in TB-free areas in Vorarlberg were preceded by sporadic cases. This finding contrasts with the situation in the north of Vorarlberg, where sporadic cases have been recorded for nine years without any indication of spread among the local deer population. Deer abundance and management and size of winter feeding sites are comparable in both the TB control area and the area with sporadic cases, and deer densities are estimated to be high enough to support spread in the resident deer population. The north of Vorarlberg is part of the foothills of the alps with lower mountains and better feed availability for deer. Deer are on average heavier and might thus be in a better physical condition compared to deer in the TB control area. However, it is questionable if this physical advantage is sufficient to prevent the establishment of a new TB spot given that the environmental conditions in the north of Vorarlberg resemble those in the TB areas in nearby southern Germany.

Sporadic cases would be expected in various disease scenarios: they could either indicate that TB became established at a very low level in resident deer and is therefore constantly present—or they could be an indicator for a nearby active TB situation from where cases “spill over” into new areas. For the north of Vorarlberg, the results of the deer monitoring rather support the latter, with regular introductions of infected migratory deer from the TB control area in Vorarlberg or neighboring deer TB areas in Tyrol or Germany. Even in the “TB at a low level” scenario deer with advanced lesions of score 4 or 5 would occasionally spend the winter at feeding sites together with critical numbers of susceptibles. This should eventually lead to the detection of additional cases in resident deer.

Sick deer are likely to isolate themselves from their social group. It could be observed that deer with severe TB were commonly found alone and well away from other deer (12). If sporadic cases seen in the north of Vorarlberg are such isolated deer that have migrated from affected areas, this means that even advanced TB is no obstacle for diseased animals to move over long distances. The pattern of sporadic cases being mostly older stags is consistent with results from a telemetry study in the south of Vorarlberg: this study showed that stags more often migrate over long distances up to 30 km across mountains and have a larger mean home range size of 6,400 hectares, compared to females with 2,600 hectares (35). The kill sites of sporadic cases were in distances between 1 and 13 km from the borders to Tyrol, Germany and the TB control area of Vorarlberg. The origin of migratory deer can therefore not be determined based merely on the distance of the kill site to the closest TB area. Cross-border cooperation is needed to better understand the dynamics of TB in this border area. Comparative genomic analyses could provide insights into the relationship of mycobacteria circulating in the different affected regions.

### Lesion Presence as Indicator for Disease Progress

At individual animal-level, environmental factors as well as animal-specific factors are understood to influence progression of TB and other diseases within the infected body (20, 21). This implies that the time period to progress from one stage of lesions to the next might vary considerably between infected individuals.

Latency is an important characteristic of *M. tuberculosis* infection in humans (49, 50) described it also as the most frequent expression of *M. bovis* infection in badgers. In cattle, latent TB infections are not considered to be common (51), and *M. bovis* infection of cattle usually results in a slowly progressive disease (52). For red deer, it is not known how often latent TB infections occur and which role they play in infection dynamics. Studies on *M. caprae* in red deer in Austria (4) and Germany (2) and *M. bovis* in red deer in Spain (44, 53) and New Zealand (8, 12, 19) showed that the pathogen could be detected in 22–68% of deer samples without visible lesions. Gavier-Widen et al. (54) argued that this non-visible lesion presentation in animals was likely to include latent cases or merely early-stage infections that do not yet present macroscopic lesions. However, it is uncertain if animals with non-visible lesions would eventually develop progressive disease or if these infections can be cured spontaneously (52, 54). Until host immune mechanisms of red deer are not better understood, inferences on the potential time of TB infection based on lesion need to be made with caution. Especially with adult deer, score 1 lesions cannot be put on a level with recent infections without any supporting epidemiological data. However, lack of gross lesions in the total of 351 tested fawns till December in combination with presence of small lesions detected from April onwards in yearlings of the same birth cohort indicate that these score 1 lesions indeed correspond to recent infections that potentially occurred during the winter feeding period.

Being aware of these open questions, results of this work nevertheless support the approach that an analysis of tissue lesions at population-level is still useful to monitor developments in infection dynamics. The Patho Score allows visualization and quantification of these dynamics. The Patho Score presented in this study focuses on lesions in medial retropharyngeal lymph nodes and discriminates particularly between mild to moderate stages of lesions. At the population level these lesions generally indicate recent infections (11, 33). Predominance of lesions in medial retropharyngeal lymph nodes reported in this study was previously described for *M. caprae* in Austrian red deer by Fink et al. (5) and Nigsch (22). This observation corresponds to results of studies from other countries (11, 15, 53) and supports the conclusion that monitoring programs that focus on the examination of lymphoid tissue of the head are capable to detect a significant portion of TB-infected red deer (53) and are also suitable for early detection of TB.

### Applications of the Patho Score

Lesion scores are regularly used in experimental TB challenge studies of cattle, deer and other wildlife, where tissue materials can be sampled under standardized situations (21, 55–57). With naturally infected deer, lesion scores were applied to study infection patterns and effects of TB on deer, to assess the role of deer in perpetuating TB among cattle or to develop sampling protocols (17, 19, 53). In this study, the Patho Score was used to identify risk groups for advanced TB stages and to characterize areas with different patterns of disease occurrence. For many analyses, such as detailed analyses of outbreaks or analyses of sporadic cases, the absolute number of TB cases was too low to obtain statistically significant results. These mainly descriptive analyses are thus considered as exploratory. One strength of the Patho Score is certainly that with the descriptive information it generates, it provides a much more differentiated insight into the TB situation compared to prevalence data alone, at no extra costs. This information can then be used for forming hypotheses to be investigated via more rigorous, multivariable statistical methods in a next step, and for guiding early disease management efforts until those hypotheses are validated.

The overarching goal of deer monitoring is to protect cattle against TB infections from deer. For Vorarlberg, no studies are available on the interaction between cattle and deer, but the main route of infection is assumed to be indirect transmission. The Patho Score helps to identify areas with an increased risk of environmental contamination by deer with advanced stages of TB, or areas where the risk of infection could increase rapidly due to recent outbreaks in deer. Identification of these high-risk areas is an important prerequisite for targeted measures toward disease prevention in cattle.

Future applications of the Patho Score include comparing infection dynamics of TB in different countries or to support comparison of different monitoring systems, e.g., how successful is the monitoring system to detect very small TB lesions.

### Limitations

Deer monitoring and sampling are conducted under field conditions: by definition, the hunting bag is not a simple random sample of the local deer population. On the one hand, some age groups are underrepresented in the hunting bag as red deer management favors a specific age pyramid. On the other hand, the hunting law foresees that obviously sick and injured animals must be harvested for welfare purposes. In the TB control area all sick and injured deer had to be examined and were thus likely to be overrepresented in the sample.

In addition, only tissue material with gross lesions was selected for further examinations to confirm TB. Tissue without lesions was considered TB negative. Lesion-based monitoring tends to underestimate the prevalence. These limitations were known to the authors before the development of the Patho Score. Therefore, the challenge was to develop a valid tool under the given conditions, which is capable to take account of the heterogeneity of the available historical longitudinal data.

A critical task was to assess the impact of missing lymph nodes or other organ tissues in individual samples for the correct categorization with the Patho Score. The association between number of sample tissues and Patho Score was significant. Deer represented with thoracic or abdominal tissues in addition to heads received more frequently a high score of 4 or 5. However, submission of more tissues in addition to the standard sample (head and thorax) has only in one case led to a higher score. The reason for this lies in the definitions for score 4 and 5 (Table 1): samples with one affected organ (lung, pleura, liver, udder, etc) are categorized at least with score 4, and one severely affected body site is sufficient to receive score 5. Submission of more abnormal tissues will not necessarily increase a high score.

“Head-only” samples could by definition only reach a maximum score of 4. Sample selection in the current deer monitoring might thus underestimate the proportion of high scores and thereby the proportion of potential super-spreaders among identified cases. However, the amount of submitted tissues and severity of clinical lesions were also causally related: for deer with visible organ abnormalities, deer monitoring required that more tissues including all affected body sites were sampled. The potential bias in selection of sample material was accounted for twofold: firstly, by adjusting for the amount of sample material in the statistical analysis, and secondly by the final interpretation of the Patho Score: in this study, scores 4 and 5 were both interpreted to be more relevant for spreading disease, with score 5 being considered as an advanced stage of score 4. Standardization of sample material (if logistically feasible) would have a positive effect on the overall sensitivity of deer monitoring and furthermore would increase validity of the Patho Score.

Even though deer monitoring will underestimate true prevalence, the authors hypothesize that comparative analyses over time and space remain valid, as sampling mode and diagnostic protocol did not change greatly over the 9-year monitoring period. With lesion-based monitoring the role of animals with non-visible lesions for the infection dynamic could not be investigated. Such an investigation would require culturing of key lymph nodes from all deer, including those with non-visible lesions. However, it can be assumed that deer with gross lesions play at least for pathogen spread a more important role. With 4,521 examined samples virtually the whole range of stages of lesions could be explored. For an external evaluation of validity of the Patho Score, it would be of interest to apply this score on data from other regions to estimate the effect of field conditions.

### Recommendations

The assessment of TB-like lesions showed various practical approaches on how to gain better insight into the infection dynamics through the targeted selection of animals to be sampled in early spring to early identify new spots of infection. Identified risk groups for TB and advanced lesions should receive particular attention in infection control programs. Special attention require also sporadic cases in TB-free areas: they do not necessarily indicate that the infection already spreads locally but these sporadic cases appear to be a precursor of outbreaks among resident deer populations. In this context, one relevant question for further research would be: what constellation of animal-specific parameters, lesions, season and other measurable conditions would signal a transition of an area with sporadic cases to an outbreak area or to an area with an endemic level of infection presence?

The next step to draw a holistic picture of the infection dynamics would be to include home range size, habitat selection and deer-to-deer interaction within and between deer populations in this alpine setting in more detail to investigate potential seasonality of infections and to better characterize the role of specific subgroups in maintenance and spread of TB. Furthermore, characteristics of the pathogen should be considered in addition to host-specific, environmental and human interaction related parameters. This could be taken into account in the form of genomic analyses of infection chains between animals or between subpopulations of animals. With the ultimate goal to better understand host-pathogen interactions for this important pathogen. For this task it will be very valuable to link data generated by pathology, diagnostics, epidemiology and systems biology research.

## Conclusion

This is the first study of *M. caprae* in red deer in the Austrian state of Vorarlberg that describes development of TB and its infection dynamics over the last decade. The study proposes the use of TB-like lesions in a so-called Patho Score as a mirror for infection dynamics. With the Patho Score, a new instrument is introduced to complement monitoring of TB in red deer in western Austria and to systematically visualize and quantify infection dynamics at no additional costs. This work shows the breadth of application possibilities of this lesion score. The analysis adds some evidence regarding the critical role of winter feeding sites for spread of TB infections in young deer. The identification of geographic areas with differing patterns of disease occurrence demonstrated that TB does spread in Vorarlberg within several geographically connected subpopulations with separate infection cycles. TB spreads only slowly between valleys but migrating infected deer might introduce the agent into new areas.

To the best knowledge of the authors, this is the first study that uses a lesion score for the systematical description of the infection dynamics of mycobacterial disease. Due to the cross-border TB situation, the possibility to systematically compare TB dynamics based on heterogeneous data is an important added value.

## Data Availability Statement

Restrictions apply to the datasets: The raw data for this manuscript are not publicly available and are subject to a data protection clause. Requests to access the datasets will be examined on a case to case basis and should be directed to Norbert Greber, veterinaer@vorarlberg.at. Aggregated data supporting the conclusions of this manuscript is contained within the manuscript and the Supplementary Files.

## Ethics Statement

All animal sampling was post-mortem in accordance with the official deer monitoring program plan of the Office of the State Government of Vorarlberg. Wildlife samples came from hunter-harvested individuals that were shot during the legal hunting season, or individuals found dead, independently and prior to our research. According to national legislation (Austrian Tierversuchsgesetz 2012 – TVG 2012, BGBl. I Nr. 114/2012) no permission or consent was required for conducting this type of study.

## Author Contributions

AN, WG, and NG designed the study. WG and ZB carried out the laboratory work. WG and NG entered and prepared the data for the analysis. AN, WG, and ZB developed the Patho Score. AN performed the analysis. AN, WG, and ZB drafted the preliminary manuscript. All authors participated in the review and the editing of the draft and approved its final version.

### Conflict of Interest Statement

The authors declare that the research was conducted in the absence of any commercial or financial relationships that could be construed as a potential conflict of interest.
