# Supplementary material for: Mycobacterium caprae Infection of Red Deer in Western Austria–Optimized Use of Pathology Data to Infer Infection Dynamics
Source: Front Vet Sci. 2019 Jan 21;5:350. doi: 10.3389/fvets.2018.00350 (PMC6348259; doi:10.3389/fvets.2018.00350)
Supplement: Supplementary file 1 [file Data_Sheet_1.docx]

*Mycobacterium caprae* infection of red deer in western Austria – optimized use of pathology data to infer infection dynamics

# Supplementary Material


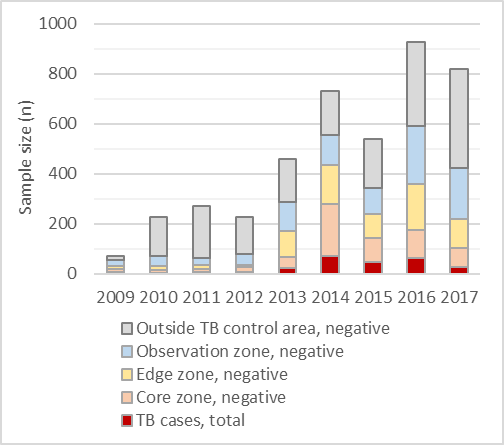


Figure S1: Sample size of TB-tested deer, per year and zone (n = 4,265). Outside TB control area, negative (n = 1,801), observation zone (n = 915), edge zone (n = 710), core zone (n = 582), TB cases within and outside the TB control area, total (n = 257).


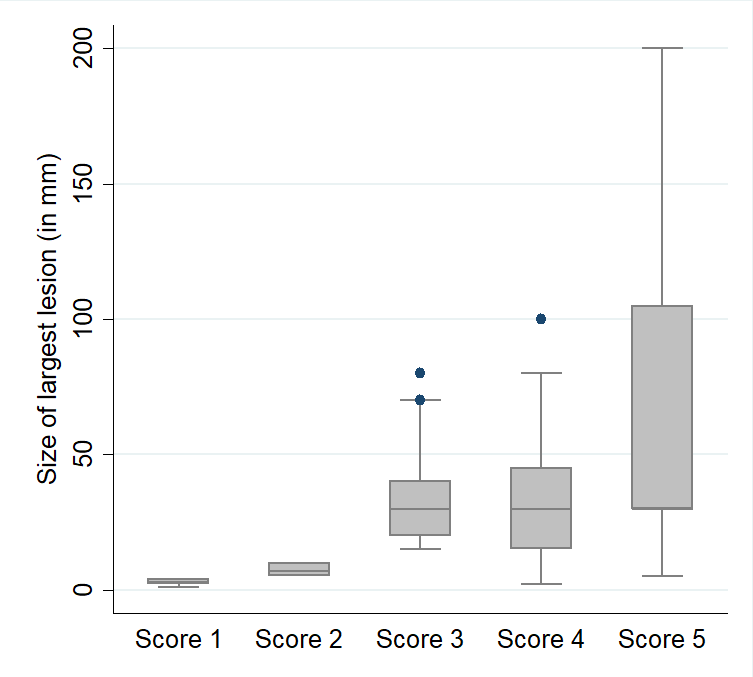


Figure S2: Size of the largest lesion (in millimeter) detected in tissue of TB positive deer (n = 256), by Patho Score level.

**Table S1: Description of the study population (n = 4,265).** Numbers of deer tested between February 2009–March 2018, by sex, age group, condition, number of submitted sample tissues and TB zone of kill location. Column “TB positive (%)” was calculated by dividing column “TB positive, total” by column “Tested, total”.

| **Subgroup** | **Score 1** | **Score 2** | **Score 3** | **Score 4** | **Score 5** | **TB positive, total** | **TB positive (%)** | **Tested, total** |
| --- | --- | --- | --- | --- | --- | --- | --- | --- |
| **Sex**^a^ |  |  |  |  |  |  |  |  |
| Female | 27 | 33 | 35 | 19 | 7 | 121 | 5.6 | 2,170 |
| Male | 21 | 24 | 36 | 35 | 19 | 136^b^ | 7.8 | 1,742 |
| **Age group, by sex** | | | | | | | | |
| Fawn^a^ | - | - | - | - | - | 0 | - | 351 |
| Female yearling | 10 | 7 | 8 | 8 | 3 | 36 | 6.0 | 599 |
| Hind (≥ 2 yrs) | 17 | 26 | 27 | 11 | 4 | 85 | 5.4 | 1,571 |
| Male yearling | 6 | 1 | 7 | 6 | 1 | 21 | 6.1 | 347 |
| Stag III (2–4 yrs) | 9 | 12 | 19 | 19 | 6 | 65 | 7.1 | 915 |
| Stag II (5–9 yrs) | 5 | 8 | 6 | 8 | 9 | 37^b^ | 16.7 | 222 |
| Stag I (≥ 10 yrs) | 1 | 3 | 4 | 2 | 3 | 13 | 5.1 | 257 |
| **Condition** | | | | | | | | |
| Good | 46 | 54 | 65 | 38 | 9 | 212 | 5.2 | 4,092 |
| Poor | 2 | 3 | 6 | 16 | 17 | 45^b^ | 26.2 | 172 |
| **Sample tissue** | | | | | | | | |
| Head-only | 32 | 28 | 34 | 23 | - | 117 | 8.6 | 1,361 |
| Additional tissues^c^ | 16 | 29 | 37 | 31 | 26 | 140^b^ | 4.8 | 2,904 |
| **Zone** | | | | | | | | |
| Core zone | 24 | 36 | 34 | 33 | 9 | 136 | 18.9 | 718 |
| Edge zone | 17 | 12 | 19 | 12 | 4 | 64 | 8.3 | 774 |
| Observation zone | 7 | 6 | 15 | 4 | 10 | 43^b^ | 4.5 | 958 |
| Zone outside TB control area | - | 3 | 3 | 5 | 3 | 14 | 0.8 | 1,815 |
| **Total** | **48** | **57** | **71** | **54** | **26** | **257**^b^ | **6.0** | **4,265**^d^ |

^a^Sex was not recorded for fawns.

^b^Data on submitted material was missing for one stag II, therefore no score was assigned.

^c^”Head and thorax”, “head, thorax and abdomen”, “thorax-only” and “other” samples.

^d^Numbers in the last column do not add up to 4,265 for all groups due to missing information for some negative animals.

Table S2: Apparent prevalences and their development in the TB control area over 2009–2017, by TB zone. Statistical output is reported in four separate tables A–D.

**Table S2_A:** Core zone – apparent prevalence, standard error and unadjusted 95% confidence interval.

over : year

--------------------------------------------------------------

| Unadjusted

core zone | Mean Std. Err. [95% Conf. Interval]

-------------+------------------------------------------------

year |

2009 | .0833333 .1125943 .0 .3043915

2010 | .0909091 .1176009 .0 .3217969

2011 | .2857143 .1042421 .0810542 .4903744

2012 | .1818182 .0831564 .0185559 .3450805

2013 | .2758621 .0512145 .1753118 .3764123

2014 | .2159091 .0240052 .1687793 .2630389

2015 | .2283465 .0346103 .1603955 .2962974

2016 | .12 .0348861 .0515076 .1884924

2017 | .1058824 .0423056 .0228231 .1889416

--------------------------------------------------------------

**Table S2_B:** Core zone – pairwise comparisons of means with equal variances.

-------------------------------------------------------------------------------

| Unadjusted Unadjusted

core zone | Contrast Std. Err. t P>|t| [95% Conf. Interval]

--------------+----------------------------------------------------------------

year |

2010 vs 2009 | .0075758 .1628111 0.05 0.963 -.3120739 .3272254

2011 vs 2009 | .202381 .1534402 1.32 0.188 -.0988706 .5036325

2012 vs 2009 | .0984848 .1399731 0.70 0.482 -.1763266 .3732963

2013 vs 2009 | .1925287 .1236948 1.56 0.120 -.0503232 .4353807

2014 vs 2009 | .1325758 .1151249 1.15 0.250 -.0934507 .3586022

2015 vs 2009 | .1450131 .1177937 1.23 0.219 -.0862531 .3762793

2016 vs 2009 | .0366667 .117875 0.31 0.756 -.1947592 .2680925

2017 vs 2009 | .022549 .1202799 0.19 0.851 -.2135983 .2586963

2011 vs 2010 | .1948052 .1571509 1.24 0.216 -.1137316 .5033419

2012 vs 2010 | .0909091 .1440312 0.63 0.528 -.1918695 .3736877

2013 vs 2010 | .184953 .1282689 1.44 0.150 -.0668793 .4367853

2014 vs 2010 | .125 .120026 1.04 0.298 -.1106488 .3606488

2015 vs 2010 | .1374374 .1225881 1.12 0.263 -.1032419 .3781166

2016 vs 2010 | .0290909 .1226663 0.24 0.813 -.2117417 .2699236

2017 vs 2010 | .0149733 .124979 0.12 0.905 -.2303999 .2603464

2012 vs 2011 | -.1038961 .1333469 -0.78 0.436 -.3656982 .157906

2013 vs 2011 | -.0098522 .1161436 -0.08 0.932 -.2378788 .2181744

2014 vs 2011 | -.0698052 .1069704 -0.65 0.514 -.2798218 .1402114

2015 vs 2011 | -.0573678 .1098375 -0.52 0.602 -.2730136 .1582779

2016 vs 2011 | -.1657143 .1099248 -1.51 0.132 -.3815313 .0501027

2017 vs 2011 | -.1798319 .1124997 -1.60 0.110 -.4007043 .0410404

2013 vs 2012 | .0940439 .0976623 0.96 0.336 -.097698 .2857857

2014 vs 2012 | .0340909 .086552 0.39 0.694 -.1358379 .2040197

2015 vs 2012 | .0465283 .0900714 0.52 0.606 -.1303104 .2233669

2016 vs 2012 | -.0618182 .0901778 -0.69 0.493 -.2388656 .1152292

2017 vs 2012 | -.0759358 .0932993 -0.81 0.416 -.2591117 .1072401

2014 vs 2013 | -.059953 .0565613 -1.06 0.290 -.1710006 .0510946

2015 vs 2013 | -.0475156 .0618126 -0.77 0.442 -.1688733 .0738421

2016 vs 2013 | -.1558621 .0619675 -2.52 0.012 -.2775237 -.0342004

2017 vs 2013 | -.1699797 .0664281 -2.56 0.011 -.300399 -.0395604

2015 vs 2014 | .0124374 .0421203 0.30 0.768 -.0702581 .0951329

2016 vs 2014 | -.0959091 .0423472 -2.26 0.024 -.1790501 -.0127681

2017 vs 2014 | -.1100267 .0486417 -2.26 0.024 -.2055257 -.0145278

2016 vs 2015 | -.1083465 .0491417 -2.20 0.028 -.2048272 -.0118657

2017 vs 2015 | -.1224641 .0546593 -2.24 0.025 -.2297775 -.0151507

2017 vs 2016 | -.0141176 .0548343 -0.26 0.797 -.1217747 .0935394

-------------------------------------------------------------------------------

**Table S2_C:** Edge and observation zones (joined) – apparent prevalence, standard error and unadjusted 95% confidence interval.

over : year

--------------------------------------------------------------

| Unadjusted

E_O_zone | Mean Std. Err. [95% Conf. Interval]

-------------+------------------------------------------------

year |

2009 | .1190476 .03698 .0465173 .191578

2010 | .0175439 .0317434 .0 .0798036

2011 | .0625 .0345916 .0 .1303459

2012 | .0363636 .0323154 .0 .0997452

2013 | .0266667 .0159772 .00467 .0580034

2014 | .0448276 .0140732 .0172253 .0724299

2015 | .0657277 .0164211 .0335204 .097935

2016 | .1008584 .0111019 .0790837 .122633

2017 | .047619 .0130744 .0219757 .0732624

--------------------------------------------------------------

**Table S2_D:** Edge and observation zones (joined) – pairwise comparisons of means with equal variances.

-------------------------------------------------------------------------------

| Unadjusted Unadjusted

E_P_zone | Contrast Std. Err. t P>|t| [95% Conf. Interval]

--------------+----------------------------------------------------------------

year |

2010 vs 2009 | -.1015038 .0487356 -2.08 0.037 -.197091 -.0059165

2011 vs 2009 | -.0565476 .0506369 -1.12 0.264 -.1558639 .0427687

2012 vs 2009 | -.082684 .0491101 -1.68 0.092 -.1790057 .0136378

2013 vs 2009 | -.092381 .0402838 -2.29 0.022 -.1713913 -.0133706

2014 vs 2009 | -.07422 .0395673 -1.88 0.061 -.1518251 .003385

2015 vs 2009 | -.0533199 .0404619 -1.32 0.188 -.1326796 .0260398

2016 vs 2009 | -.0181892 .0386105 -0.47 0.638 -.0939176 .0575391

2017 vs 2009 | -.0714286 .0392232 -1.82 0.069 -.1483586 .0055015

2011 vs 2010 | .0449561 .0469492 0.96 0.338 -.0471272 .1370395

2012 vs 2010 | .0188198 .0452982 0.42 0.678 -.0700256 .1076651

2013 vs 2010 | .0091228 .0355375 0.26 0.797 -.0605784 .078824

2014 vs 2010 | .0272837 .0347232 0.79 0.432 -.0408203 .0953877

2015 vs 2010 | .0481838 .0357393 1.35 0.178 -.0219131 .1182808

2016 vs 2010 | .0833145 .0336288 2.48 0.013 .0173569 .1492721

2017 vs 2010 | .0300752 .0343305 0.88 0.381 -.0372587 .0974091

2012 vs 2011 | -.0261364 .0473378 -0.55 0.581 -.1189819 .0667092

2013 vs 2011 | -.0358333 .0381031 -0.94 0.347 -.1105666 .0388999

2014 vs 2011 | -.0176724 .0373448 -0.47 0.636 -.0909183 .0555735

2015 vs 2011 | .0032277 .0382914 0.08 0.933 -.0718748 .0783302

2016 vs 2011 | .0383584 .0363295 1.06 0.291 -.0328961 .1096129

2017 vs 2011 | -.014881 .03698 -0.40 0.687 -.0874113 .0576494

2013 vs 2012 | -.009697 .0360494 -0.27 0.788 -.0804021 .0610081

2014 vs 2012 | .0084639 .0352469 0.24 0.810 -.0606672 .0775951

2015 vs 2012 | .0293641 .0362483 0.81 0.418 -.0417312 .1004593

2016 vs 2012 | .0644947 .0341693 1.89 0.059 -.0025229 .1315123

2017 vs 2012 | .0112554 .0348601 0.32 0.747 -.0571171 .079628

2014 vs 2013 | .0181609 .0212914 0.85 0.394 -.0235988 .0599207

2015 vs 2013 | .039061 .0229112 1.70 0.088 -.0058756 .0839977

2016 vs 2013 | .0741917 .0194557 3.81 0.000 .0360325 .1123509

2017 vs 2013 | .0209524 .0206448 1.01 0.310 -.0195392 .061444

2015 vs 2014 | .0209001 .0216265 0.97 0.334 -.0215169 .0633171

2016 vs 2014 | .0560308 .017925 3.13 0.002 .0208737 .0911879

2017 vs 2014 | .0027915 .0192092 0.15 0.884 -.0348844 .0404673

2016 vs 2015 | .0351307 .0198218 1.77 0.077 -.0037467 .074008

2017 vs 2015 | -.0181087 .0209903 -0.86 0.388 -.0592777 .0230604

2017 vs 2016 | -.0532393 .017152 -3.10 0.002 -.0868803 -.0195983

-------------------------------------------------------------------------------

Table S3: Supporting data table to Figure 6 with age group, kill date and Patho Score. Identifiers in the first column match with case numbers in Figure 6.

| **Case ID** | **Age group** | **Kill date** | **Patho Score** |
| --- | --- | --- | --- |
| 1 | Stag I | Aug-09 | 3 |
| 2 | Stag III | Sep-10 | 3 |
| 3 | Stag II | Sep-10 | 5 |
| 4 | Stag I | Nov-11 | 4 |
| 5 | Stag II | Oct-12 | 2 |
| 6 | Stag II | Oct-13 | 5 |
| 7 | Hind | Aug-14 | 2 |
| 8 | Stag I | Oct-15 | 5 |
| 9 | Stag III | Nov-15 | 3 |
| 10 | Hind | Nov-15 | 4 |
| 11 | Female yearling | Nov-16 | 4 |
| 12 | Stag I | Sep-17 | 2 |
